# Excessive activation of the TLR9/TGF-β1/PDGF-B pathway in the peripheral blood of patients with systemic lupus erythematosus

**DOI:** 10.1186/s13075-017-1238-8

**Published:** 2017-03-29

**Authors:** Yi Yuan, Mingyue Yang, Kuo Wang, Jing Sun, Lili Song, Xue Diao, Zhenyu Jiang, Genhong Cheng, Xiaosong Wang

**Affiliations:** 1 Institute of Translational Medicine, the First Hospital, Jilin University, Changchun, 130061 China; 2 Department of Rheumatology and Immunology, the First Hospital, Jilin University, Changchun, 130021 China; 3 Shanghai Wisdom Chemical Research Co. Ltd., Shanghai, 201203 China; 4 Department of Microbiology, Immunology and Molecular Genetics, University of California Los Angeles, Los Angeles, CA 90095 USA

**Keywords:** Systemic lupus erythematosus, Toll-like receptor 9, Lupus nephritis

## Abstract

**Background:**

Our aim is to study the existence of the TLR9/TGF-β1/PDGF-B pathway in healthy humans and patients with systemic lupus erythematosus (SLE), and to explore its possible involvement in the pathogenesis of lupus nephritis (LN).

**Methods:**

Protein levels of the cytokines were detected by ELISA. mRNA levels of the cytokines were analyzed by real-time PCR. MTT assay was used to test the proliferation of mesangial cells under different treatments.

**Results:**

Compared to healthy controls (*N*_Control_ = 56), levels of Toll-like receptor (TLR)9, transforming growth factor (TGF)-β1, and platelet-derived growth factor B (PDGF-B) were increased significantly in the peripheral blood of SLE patients (*N*_SLE_ = 112). Significant correlations between the levels of TLR9, TGF-β1, and PDGF-B were observed in both healthy controls and SLE patients. The levels of TGF-β1 and PDGF-B were greatly enhanced by TLR9 activation in primary cell cultures. The proliferation of mesangial cells induced by the plasma of SLE patients was significantly higher than that induced by healthy controls; PDGF-B was involved in this process. The protein levels of PDGF-B homodimer correlated with the levels of urine protein in SLE patients with LN (*N*_LN_ =38).

**Conclusions:**

The TLR9/TGF-β1/PDGF-B pathway exists in humans and can be excessively activated in SLE patients. High levels of PDGF-B may result in overproliferation of mesangial cells in the kidney that are involved in the development of glomerulonephritis and LN. Further studies are necessary to identify TLR9, TGF-β1, and PDGF-B as new therapeutic targets to prevent the development of glomerulonephritis and LN.

**Electronic supplementary material:**

The online version of this article (doi:10.1186/s13075-017-1238-8) contains supplementary material, which is available to authorized users.

## Background

Toll-like receptor (TLR)9 is expressed by a number of different immune cell types [1, 2]. It recognizes hypomethylated CpG oligonucleotide-motif DNA (CpG) in bacterial genomes and signals potent inflammatory responses. Our previously published study has identified the TLR9/TGF-β1/PDGF-B pathway as a novel signaling cascade in mouse bone marrow macrophages. It includes TLR9-mediated signal leading to the induction of platelet-derived growth factor B (PDGF-B) through the transforming growth factor (TGF)-β1 auto/paracrine loop [3].

TLR9 plays crucial roles in the pathogenesis of glomerulonephritis [4–6], though some recent studies report that TLR9 can be protective in systemic lupus erythematosus (SLE) patients [7, 8]. TGF-β1 and PDGF-B are important mediators of extracellular matrix (ECM) accumulation, fibrosis, and mesangial cell proliferation in glomerulonephritis [9–11]. It is reported that PDGF-B homodimer activates mesangial cells to proliferate and mediate glomerulosclerosis through matrix production and transdifferentiation into myofibroblasts [12]. We reported previously that the TLR9-mediated cascade to the induction of PDGF-B is critical to the promotion of mesangial cell proliferation [3]. All these results suggest a possible mechanism of glomerulonephritis in mice: the activation of TLR9 leads to the secretion of TGF-β1 and PDGF-B, which induces the production of ECM components as well as mesangial cell proliferation, migration, and accumulation, and finally leads to glomerulonephritis.

SLE is a systemic autoimmune disease characterized by the presence of autoantibodies and immune complexes that target multiple organ systems [13]. SLE poses significant challenges in diagnosis and treatment. In-depth understanding of the molecular mechanism of the disease will lead to identifying diagnosis biomarkers and the development of effective immunotherapy with relevance to the molecules involved in the pathogenesis of the disease. TLR9 is assumed to be related to the etiology of SLE in the recognition of anti-DNA antibody containing immune complexes [14, 15]. The expression of TLR9 in patients with active SLE is found to be significantly higher than in patients with inactive SLE [16], and it decreases significantly after treatment [17]. Lupus nephritis (LN) is a common manifestation of SLE which is a major cause of renal insufficiency [18, 19]. It is believed that LN is induced by autoantibody and immune complex deposition [20]. However, its etiology and pathogenic mechanisms have not been clearly elucidated [21].

It would be revealing to investigate if the TLR9/TGF-β1/PDGF-B pathway exists in humans as well, which to our knowledge has not been performed to date. In the current study, we therefore aimed at exploring the presence of the TLR9/TGF-β1/PDGF-B pathway in humans, and comparing the activation of the TLR9/TGF-β1/PDGF-B pathway between SLE patients and healthy controls. Furthermore, we explored the possible involvement of the upregulated PDGF-B in SLE patients in the increased mesangial cell proliferation and the pathogenesis of glomerulonephritis.

## Methods

### Patients and sample preparations

Blood samples and clinical indexes from 112 patients with SLE (see Additional file 1: Table S1) were collected at the Department of Rheumatology and Immunology, the first hospital of Jilin University. All patients fulfilled at least four of the 1997 revised American College of Rheumatology (ACR) classifications for SLE and did not present symptoms of active infection or neoplastic disease at the time of the study. Immunosuppressive treatment consisted of glucocorticoid plus hydroxychloroquine (HCQ); some of the patients also received mycophenolate mofelil (MMF) or azathioprine according to their different clinical manifestations. Age- and sex-matched blood samples from 56 healthy people were collected as the experiment control (see Additional file 1: Table S1); 38 SLE patients with LN were included in this study. LN was defined by the ACR lupus classification criteria as proteinuria >0.5 g per day or urinary protein higher than 3+ by dipstick analysis [22]. The study protocols and consent forms were approved by the Institutional Medical Ethics Review Board of the first hospital of Jilin University, in compliance with the Declaration of Helsinki.

Blood samples were centrifuged at 3000 rpm for 10 min at room temperature and the obtained plasma was stored at –20 °C. TRIzol reagent (1 ml; Invitrogen, Carlsbad, CA, USA) was added to 150 μl whole blood cells and stored at −80 °C.

### Enzyme-linked immunosorbent assay (ELISA)

The concentrations of TGF-β1 (eBioscience) and PDGF-B (eBioscience) were measured using ELISA kits according to the manufacturers’ instructions. The ELISA kit used for protein detection of PDGF-B is specific for PDGF-BB, which is a homodimer of PDGF-B.

For TGF-β1, samples were prepared before the test procedure. Plasma or cell culture supernatant samples were diluted 1:10 with assay buffer (1×) according to the following scheme: 20 μl sample + 180 μl assay buffer (1×). HCI (20 μl 1 N) was added to 200 μl prediluted sample, mixed, and incubated for 1 h at room temperature. Samples were neutralized by the addition of NaOH (20 μl 1 N). Human TGF-β1 standard dilutions ranging from 31 to 2000 pg/ml were created. The plate was washed twice with approximately 400 μl wash buffer per well before adding samples, and then 100 μl of standard dilutions were added to the standard wells: 100 μl of assay buffer (1×) was added to the blank wells, and 60 μl of assay buffer (1×) and 40 μl of pretreated sample were added to the sample wells.

For PDGF-B, samples were prediluted 1:10 with assay buffer (1×) according to the following scheme: 20 μl sample + 180 μl assay buffer (1×). The plate was washed twice with approximately 400 μl wash buffer per well. Human PDGF-B standard dilutions were created ranging from 31 to 2000 pg/ml, and 100 μl of standard dilutions were added to the standard wells: 100 μl of assay buffer (1×) was added to the blank wells, and 50 μl of assay buffer (1×) and 50 μl of prediluted samples were added to the sample wells.

For both TGF-β1 and PDGF-B, each sample, standard, and blank was assayed in duplicate. After adding the samples, plate was incubated at room temperature for 2 h on a microplate shaker set at 400 rpm. Next, the plate was washed and incubated with biotin-conjugate, streptavidin-HRP and TMB substrate solution in sequential order following the instructions. When the highest standard had developed a dark blue color, 100 μl stop solution was added. Absorbance of the plate was read on the Synergy H1 Hybrid Reader (Biotek, Winooski, VT, USA) using 450 nm as the primary wavelength. A standard curve was generated from the readings of the diluted standards. Sample concentrations were calculated based on their absorbance compared to the standard curve. All of the samples were tested twice and the results were averaged.

### Real-time quantitative polymerase chain reaction (qPCR)

Total RNA was extracted from whole blood cells or isolated monocytes using TRIzol reagent (Invitrogen, Carlsbad, CA, USA). The purity of RNA was determined by absorbance at 260 nm and 280 nm on the Synergy H1 Hybrid Reader (Biotek, Winooski, VT, USA). The integrity of RNA was monitored by analyzing the intensity of ribosomal 18S and 28S RNA with an Agilent 2100 bioanalyser (Agilent Techonlogies, Santa Clara, CA, USA). Extracted RNA (1 μg) was reverse-transcribed using the PrimeScript RT Reagent Kit (TAKARA, Kyoto, Japan). Diluted cDNA (1:20; 5 μl) was amplified using the Fast Start Universal SYBR Green Master (Roche Diagnostics GmbH, Mannheim, Germany) and quantitative PCR using specific primers (synthesized by Sangon Biotech, Shanghai, China). The qPCR assays were carried out in duplicate on the Applied Biosystems Step one plus instrument (Step one software 2.2). The cycling conditions were 10 min polymerase activation at 95 °C, followed by 40 cycles at 95 °C for 10 s and 60 °C for 30 s. The threshold was set above the nontemplate control background and within the linear phase of the target gene amplification to calculate the cycle number at which the transcript was detected (Ct) [23]. In all of our experiments, each sample was tested twice; every Ct value was the average of the results from two wells. Glyceraldehyde-3-phosphate dehydrogenase (GAPDH) was selected as the reference gene. The method of 2^–ΔΔCT^ was used to analyze the real-time PCR data expressed as the fold-change relative to the average value of the GAPDH [24, 25].

### Cell culture and treatment

Whole blood samples were diluted at 1:10 in the RPMI 1640 medium with 100 units/ml penicillin and 100 μg/ml streptomycin at 37 °C in 5% CO_2_ atmosphere for 24 h [26, 27]. Class B CpG (ODN 2006 sequence 5′-tcgtcgttttgtcgttttgtcgtt-3′) was used as the TLR9 agonist. For Fig. 4, cells from SLE patients or healthy controls were incubated with or without CpG (Sangon Biotech) at a final concentration of 500 nM for 24 h, and then cells were harvested, lysed with TRIzol reagent, and stored at –80 °C for RNA extractions. Results are shown as the average of the triplicated wells for each sample and each treatment. For Fig. 5, cells from SLE patients were incubated with or without recombinant human TGF-β1 (final concentration 2.5 ng/ml; R&D Systems, Minneapolis, MN, USA), SB431542 (final concentration 5 μM; Sigma-Aldrich, St. Louis, MO, USA), anti-human TGF-β1 antibody 1D11 (final concentration 1 μg/ml; R&D Systems), and CpG (final concentration 500 nM; Sangon Biotech) for 24 h. Then cells were harvested, lysed with TRIzol reagent, and stored at –80 °C for RNA extractions. Supernatants were collected and stored at –20 °C. Results are shown as the average of triplicated wells for each sample and each treatment. For Fig. 6, anti-human PDGF-B (homodimer) polyclonal antibody (R&D Systems) and isotype control antibody (R&D Systems) were added into the culture at a final concentration of 1 or 10 μg/ml; detailed information can be found in the next paragraph. For Additional file 2: Figure S1, peripheral blood mononuclear cells (PBMCs) were isolated from the peripheral blood of SLE patients by lymphoprep density-gradient centrifugation (Axis-Shield PoC AS, Oslo, Norway). Monocytes were isolated using human CD14 magnetic beads (Miltenyi Biotec; the purity was generally >95%) from PBMC for cell culture. Monocytes were incubated with or without CPG at a final concentration of 500 nM for 24 h, and then cells were harvested, lysed with TRIzol reagent, and stored at –80 °C for RNA extractions. Results are shown as the average of the triplicated wells for each sample and each treatment.

### Cell proliferation assay

Mycoplasma-free SV40 MES 13 (Murine Mesangial) cells were from the American Type Culture Collection (Manassas, VA, USA) at passage 27. Cells were maintained in Dulbecco’s modified Eagle’s medium/F12 medium (3:1) supplemented with both 14 mM HEPES (pH 7.4) and 5% fetal bovine serum (FBS). Cell monolayers were routinely grown to confluence in a humidified 37 °C, 5% CO_2_ incubator before testing. All experiments were performed between passages 30 and 40 to minimize the effects of phenotypic variation in continuous culture. Cell proliferation was measured using the 3-(4,5-dimethylthiazol-2-yl)-2,5-diphenyl-2H-tetrazolium bromide (MTT; Sigma-Aldrich) assay: 10^3^ cells/200 μl/well were seeded in a 96-well microplate and were serum starved in RPMI1640 without FBS for 24 h before treatments. For Fig. 6a, 200 μl culture medium of whole blood cells from SLE patients stimulated with CpG (500 nM) was added into starved mesangial cells and cultured for 4 h. For Fig. 6b, after supernatants were removed from starved mesangial cells, 190 μl RPMI 1640 (without FBS) and 10 μl plasma from SLE patients or healthy controls was added and cultured for 20 h. For Fig. 6c, after supernatants were removed from starved mesangial cells, 190 μl RPMI 1640 (without FBS; with or without antibodies) and 10 μl plasma from SLE patients was added and cultured for 20 h. The final concentration of anti-PDGF-B (homodimer) neutralizing antibody and control antibody were 1 or 10 μg/ml. Then, 20 μl MTT solution (5 mg/ml) was added and the incubation continued for another 4 h. Finally, the medium was carefully changed with 150 μl dimethylsulfoxide (DMSO) and the optical density was measured at 570 nm with a microplate reader. Each culture condition for each patient or control was repeated in six wells in parallel; the result is the average reading of these six wells.

### Statistical analysis

Statistical analyses were performed using Graphpad Prism 5.0 (GraphPad Software, San Diego CA, USA). Wilcoxon signed rank test for paired samples and Mann-Whitney *U* test for unpaired samples were applied. The nonparametric Spearman rank correlation test was applied for the correlation studies. *p* < 0.05 was considered statistically significant.

## Results

### Increased levels of TLR9, TGF-β1, and PDGF-B in peripheral blood of SLE patients

**Fig. 1:**
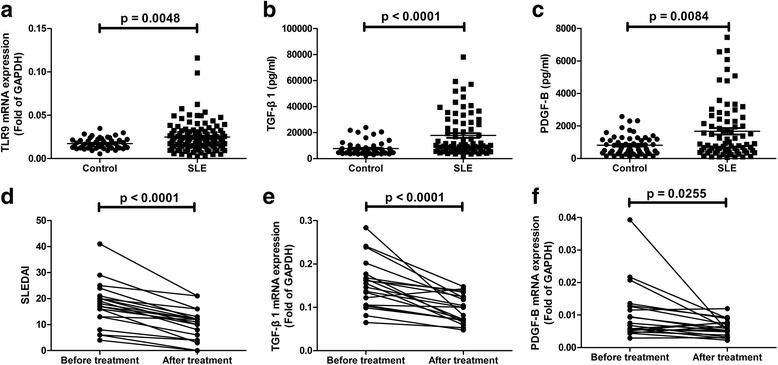
Levels of Toll-like receptor 9 (*TLR9*), transforming growth factor-β1 (*TGF-*β*1*), and platelet-derived growth factor-B (*PDGF-B*) in the peripheral blood of systemic lupus erythematosus (*SLE*) patients. The mRNA expression of TLR9 (**a**) in the peripheral blood of SLE patients (*N*_SLE_ = 112) and healthy controls (*N*_Control_ = 49). Levels of TGF-β1 (**b**) and PDGF-B (**c**) in the plasma of SLE patients (*N*_SLE_ = 77) and healthy controls (*N*_Control_ = 56). Levels of SLE Disease Activity Index (*SLEDAI*) (**d**), TGF-β1 (**e**), and PDGF-B (**f**) in the same group of SLE patients (*N*_SLE_ = 20) before and after immunosuppressive treatment. The results in a.b.c. are presented as Mean and SEM. *GAPDH* glyceraldehyde-3-phosphate dehydrogenase

First, by measuring the levels of TLR9, TGF-β1, and PDGF-B in the peripheral blood of SLE patients, we studied the possible involvement of the TLR9/TGF-β1/PDGF-B pathway in the pathogenesis of SLE. The mRNA levels of TLR9 in the blood cells of SLE patients were significantly higher than those of healthy controls (*p* = 0.0048; Fig. 1a). Furthermore, SLE patients showed much higher protein levels of TGF-β1 (*p* < 0.0001; Fig. 1b) and PDGF-B (*p* = 0.0084; Fig. 1c) compared to healthy controls. The SLE Disease Activity Index (SLEDAI) is an indicator of the disease activity of SLE. Two weeks after immunosuppressive treatment, the SLEDAI of patients decreased significantly (*p* < 0.0001; Fig. 1d), which indicates that SLE is being effectively alleviated. TGF-β1 and PDGF-B levels in the same group of patients at these two time points were compared as well. With the decrease in disease activity, levels of TGF-β1 (*p* < 0.0001; Fig. 1e) and PDGF-B (*p* = 0.0255; Fig. 1f) in SLE patients decreased greatly. These results suggest that TLR9, TGF-β1, and PDGF-B may be involved in the pathogenesis of SLE.

### Correlations among levels of TLR9, TGF-β1, and PDGF-B in peripheral blood

**Fig. 2:**
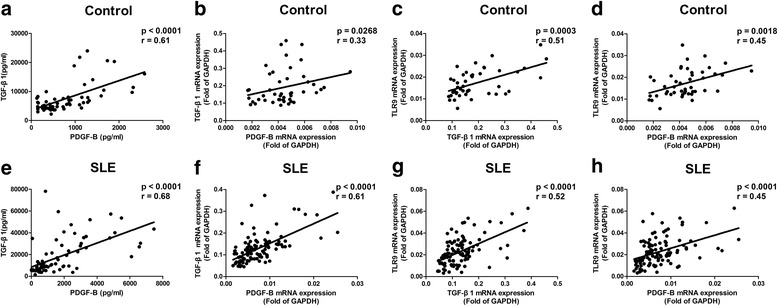
Correlations among levels of Toll-like receptor 9 (*TLR9*), transforming growth factor-β1 (*TGF-*β*1*), and platelet-derived growth factor-B (*PDGF-B*) in human peripheral blood. Correlations between protein levels of TGF-β1 and PDGF-B in healthy controls (**a**; *N*_Control_ = 56) and systemic lupus erythematosus (*SLE*) patients (**b**; *N*_SLE_ = 79). Correlations between mRNA expression of TGF-β1 and PDGF-B in healthy controls (**c**; *N*_Control_ = 46) and SLE patients (**d**; *N*_SLE_ = 106). Correlations between TLR9 and TGF-β1 (**e**,**f**), or between TLR9 and PDGF-B (**g**,**h**). The mRNA expression of healthy controls (**e**,**g**; *N*_Control_ = 46) and that of SLE patients (**f**,**h**; *N*_SLE_ = 106). *GAPDH* glyceraldehyde-3-phosphate dehydrogenase

We previously reported that the TLR9 agonist regulates PDGF-B production and cell proliferation through a TGF-β1 signal in mice [3]. To establish the relevance of these findings in humans, the relationship between levels of TLR9, TGF-β1, and PDGF-B in peripheral blood were studied. Results showed that protein levels of PDGF-B closely correlated with TGF-β1 in healthy controls (*p* < 0.0001, *r* = 0.61; Fig. 2a). Protein levels of PDGF-B correlated with TGF-β1 in SLE patients (*p* < 0.0001, *r* = 0.68; Fig. 2e) as well. Accordingly, the mRNA levels of TGF-β1 correlated closely with PDGF-B both in healthy controls (*p* = 0.0268, *r* = 0.33; Fig. 2b) and in SLE patients (*p* < 0.0001, *r* = 0.61; Fig. 2f). Furthermore, a significant correlation between mRNA levels of TLR9 and TGF-β1 (*p* = 0.0003, *r* = 0.51; Fig. 2c) and great correlation between mRNA levels of TLR9 and PDGF-B (*p* = 0.0018, *r* = 0.45; Fig. 2d) were detected in healthy controls. Moreover, it was found that mRNA levels of TLR9 were correlated with TGF-β1 (*p* < 0.0001, *r* = 0.52; Fig. 2g) and PDGF-B (*p* < 0.0001, *r* = 0.45; Fig. 2h) in SLE patients. Overall, significant correlations between the levels of TLR9, TGF-β1, and PDGF-B were observed in the blood of both SLE patients and healthy controls. These results support the hypothesis that the signal pathway of TLR9/TGF-β1/PDGF-B exists both in healthy controls and in SLE patients.

### Correlations between levels of TLR9 and MCP-1, TLR9 and ISG15, or TLR9 and IFNα

**Fig. 3:**
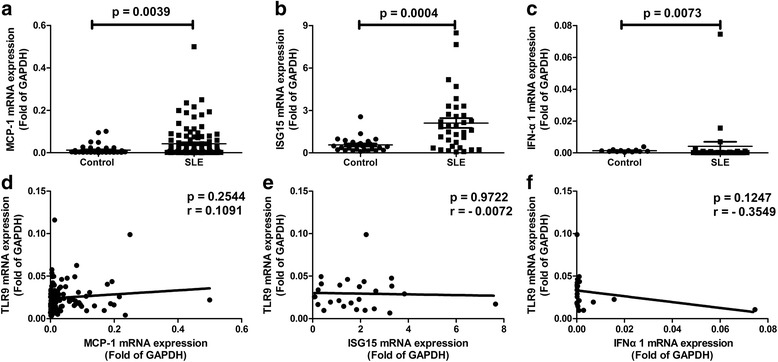
Levels of monocyte chemoattractant protein-1 (*MCP-1*), interferon-stimulated gene 15 (*ISG15*), and interferon alpha (*IFN-*α) and their correlations with Toll-like receptor 9 (*TLR9*) in systemic lupus erythematosus (*SLE*) patients. The mRNA expression of MCP-1 (**a**; *N*_Control_ = 49, *N*_SLE_ = 111), ISG15 (**b**; *N*_Control_ = 26, *N*_SLE_ = 34), and IFNα (**c**; *N*_Control_ = 12, *N*_SLE_ = 26) in the peripheral blood of SLE patients and healthy controls. The results in a.b.c are presented as Mean and SEM. Correlations between levels of TLR9 and MCP-1 (**d**; *N*_SLE_ = 111), ISG15 (**e**; *N*_SLE_ = 26), or IFNα (**f**; *N*_SLE_ = 20). *GAPDH* glyceraldehyde-3-phosphate dehydrogenase

In order to make comparisons with TLR9, TGF-β1, and PDGF-B, other cytokines (including monocyte chemoattractant protein (MCP)-1, interferon-stimulated gene (ISG)15, and interferon (IFN)α) were detected in SLE patients. The levels of MCP-1 (*p* = 0.0039; Fig. 3a), ISG15 (*p* = 0.0004; Fig. 3b), and IFNα (*p* = 0.0073; Fig. 3c) were found to increase significantly in the peripheral blood of SLE patients compared to that of healthy controls. Next, the correlations between the levels of TLR9 and theses cytokines were analyzed. It was found that, unlike TGF-β1 and PDGF-B, no significant correlation was found between TLR9 and MCP-1 (*p* = 0.2544, *r* = 0.1091; Fig. 3d), TLR9 and ISG15 (*p* = 0.9722, *r* = –0.0072; Fig. 3e), or TLR9 and IFNα (*p* = 0.1247, *r* = –0.3549; Fig. 3f).

### The mRNA expressions of TGF-β1 and PDGF-B enhanced by TLR9 activation

**Fig. 4:**
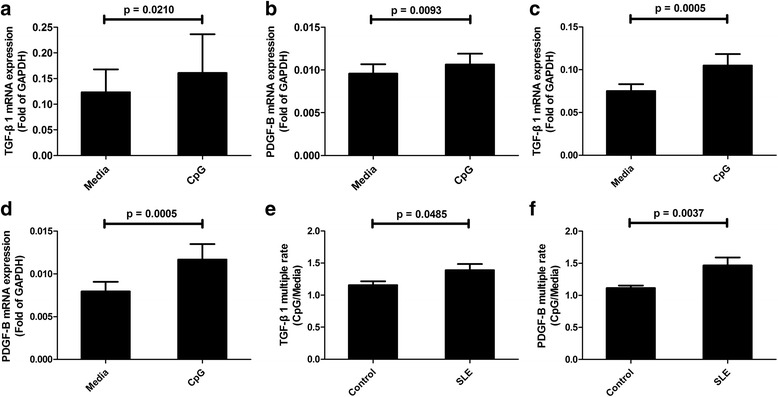
CpG induces upregulation of transforming growth factor-β1 (*TGF-*β*1*) and platelet-derived growth factor-B (*PDGF-B*) in vitro. Blood cells from healthy controls (**a**,**b**; *N*_Control_ = 12) and systemic lupus erythematosus (*SLE*) patients (**c**,**d**; *N*_SLE_ = 16) were stimulated with or without 500 nM CpG for 24 h, and then the mRNA expression of TGF-β1 and PDGF-B were detected by qPCR. Multiple rates of mRNA expression of TGF-β1 (**e**) and PDGF-B (**f**) in healthy controls (*N*_Control_ = 12) and SLE patients (*N*_SLE_ = 16) were calculated as CpG/Media. The results were presented as mean and SEM. *GAPDH* glyceraldehyde-3-phosphate dehydrogenase

We further explored the possible existence of the TLR9/TGF-β1/PDGF-B pathway in healthy humans and SLE patients in primary cell cultures. As the assay mimics the natural environment, whole blood stimulation has been used to investigate the cellular responsiveness to a variety of stimuli [28]. CpG was added to the culture as a specific stimulus for TLR9. As shown in Fig. 4, CpG can significantly upregulate the mRNA levels of TGF-β1 (*p* = 0.0210; Fig. 4a) and PDGF-B (*p* = 0.0093; Fig. 4b) in the blood cells of healthy controls and SLE patients (TGF-β1: *p* = 0.0005; Fig. 4c) (PDGF-B: *p* = 0.0005; Fig. 4d). These results support the hypothesis that the TLR9/TGF-β1/PDGF-B pathway exists in both healthy controls and SLE patients. Next, to investigate the extent of the activation of this pathway in these two groups, multiples of TGF-β1 and PDGF-B increased by CpG-stimulation in healthy humans and SLE patients were compared. It was found that CpG can induce much higher levels of TGF-β1 (*p* = 0.0485; Fig. 4e) and PDGF-B (*p* = 0.0037; Fig. 4f) in the cells from SLE patients than those from healthy controls. Similar results were also found in isolated monocytes from SLE patients and healthy controls (see Additional file 2: Figure S1). It is speculated that the TLR9/TGF-β1/PDGF-B pathway may be overactivated in SLE patients, and the overactivation is related to the increased levels of TLR9, TGF-β1, and PDGF-B shown in Fig. 1.

### PDGF-B production induced by TGF-β1

**Fig. 5:**
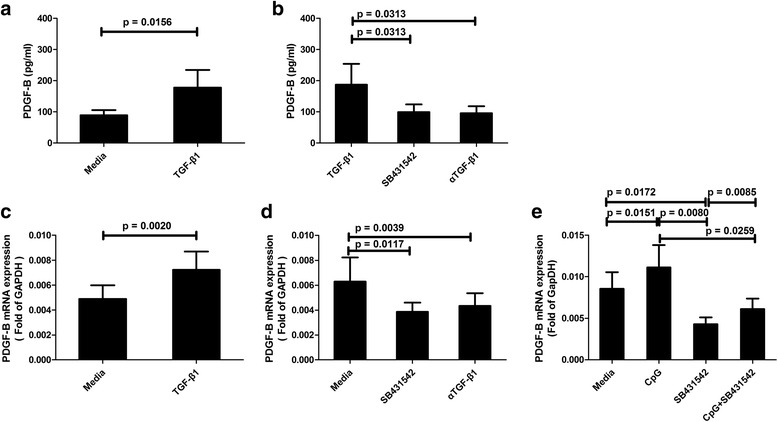
Transforming growth factor-β1 (*TGF-*β*1*) induces the production of platelet-derived growth factor-B (*PDGF-B*) in vitro. Protein levels of PDGF-B in blood cell cultures of SLE patients with or without 2.5 ng/ml recombinant protein TGF-β1 (**a**; *N*_SLE_ = 7), and with or without TGF-β1 antagonists (5 μM SB431542, 1 μg/ml αTGF-β1) (**b**; *N*_SLE_ = 6). The mRNA expression of PDGF-B in blood cell cultures of SLE patients with or without 2.5 ng/ml recombinant protein TGF-β1 (**c**; *N*_SLE_ = 10), and with or without TGF-β1 antagonists (5 μM SB431542, 1 μg/ml αTGF-β1) (**d**; *N*_SLE_ = 9). The mRNA expression of PDGF-B in blood cultures of SLE patients with 500 nM CpG, 5 μM SB431542, or 500 nM CpG plus 5 μM SB431542 (**e**; *N*_SLE_ = 16). Cells were cultured for 24 h, and then mRNA expressions were detected by qPCR. The results are presented as mean and SEM. *GAPDH* glyceraldehyde-3-phosphate dehydrogenase

To verify the role of TGF-β1 in the production of PDGF-B in SLE patients, human recombinant protein TGF-β1 was added to the culture. It was found that TGF-β1 increased protein levels (*p* = 0.0156; Fig. 5a) and mRNA levels (*p* = 0.0020; Fig. 5c) of PDGF-B significantly. On the other hand, the TGF-β1 antagonists TGF-β1 RI/ALK inhibitor SB431542 and neutralizing anti-TGF-β1 monoclonal antibody 1D11 inhibited the production of PDGF-B significantly at both the protein level (*p* < 0.05; Fig. 5b) and mRNA level (*p* < 0.05; Fig. 5d). These results suggest that TGF-β1 induces PDGF-B production in the primary peripheral blood cells of SLE patients in vitro. Moreover, SB431542 significantly inhibited the PDGF-B production induced by CpG (*p* < 0.05; Fig. 5e), indicating that TGF-β1 is greatly involved in the process of CpG-induced PDGF-B production in SLE patients. Therefore, CpG upregulates the production of PDGF-B through its induction of TGF-β1. All these results confirmed that the TLR9/TGF-β1/PDGF-B pathway exists in the peripheral blood of SLE patients.

### Higher proliferation rate of mesangial cells in SLE patients involves PDGF-B

**Fig. 6:**
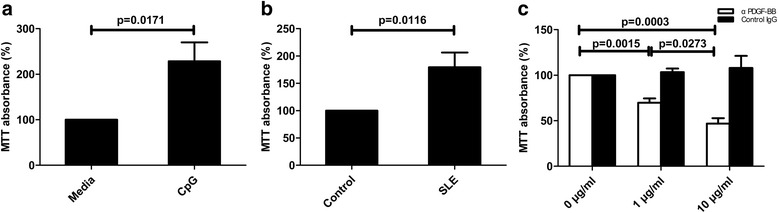
Mesangial cell proliferation induced by platelet-derived growth factor-B (*PDGF-B*) from blood cells of systemic lupus erythematosus (*SLE*) patients. Mesangial cells were serum starved for 24 h and then stimulated for 4 h with culture medium from blood cells of SLE patients (**a**; *N*_SLE_ = 8) stimulated with 500 nM CpG. Mesangial cells were serum starved for 24 h and then stimulated for 20 h with plasma from SLE patients (**b**; *N*_SLE_ = 13) or healthy controls (**b**; *N*_Control_ = 13) in culture medium. Mesangial cells were serum starved for 24 h and then stimulated for 20 h with plasma from SLE patients (**c**; *N*_SLE_ = 6), with anti-PDGF-B neutralizing antibody (1 or 10 μg/ml) or control antibody (1 or 10 μg/ml) added. Proliferation was measured by MTT assay. The results are presented as mean and SEM

PDGF-B has been recognized as a cytokine to mediate glomerulonephritis through induction of mesangial cell proliferation [10]. To determine if CpG-specific induction of PDGF-B in SLE patients affects mesangial cell proliferation, we analyzed mesangial cell proliferation by MTT assay following stimulation with culture medium from blood cells stimulated by CpG. Results showed that the supernatant of CpG-stimulated primary cells from SLE patients can potently induce mesangial cell proliferation (*p* = 0.0171; Fig. 6a). By adding plasma to the culture medium of mesangial cells and measuring the cell proliferation, it was found that plasma from SLE patients induced a much higher proliferation rate than that from healthy controls (*p* = 0.0116; Fig. 6b). Anti-PDGF-B neutralizing antibody inhibited mesangial cell proliferation induced by the plasma of patients in a dose-dependent manner (p < 0.05; Fig. 6c). These results indicate that the high levels of PDGF-B in the peripheral blood of SLE patients may have effectively induced mesangial cell proliferation and glomerulonephritis in these patients.

### PDGF-B levels of SLE patients with LN

**Fig. 7:**
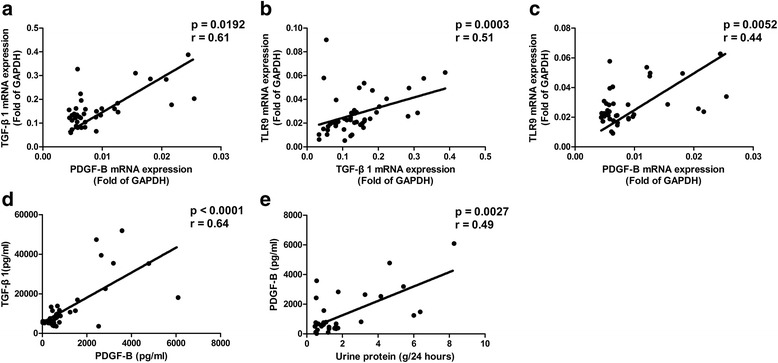
Levels of Toll-like receptor 9 (*TLR9*), transforming growth factor-β1 (*TGF-*β*1*), and platelet-derived growth factor-B (*PDGF-B*) and proteinuria in SLE patients with LN. Correlations between mRNA expression of TGF-β1 and PDGF-B (**a**; *N*_LN_ = 38). Correlations between mRNA expression of TLR9 and TGF-β1 (**b**; *N*_LN_ = 38). Correlations between mRNA expression of TLR9 and PDGF-B (**c**; *N*_LN_ = 38). Correlations between protein levels of TGF-β1 and PDGF-B (**d**; *N*_LN_ = 38). Correlations between protein levels of TGF-β1 and PDGF-B (**d**; *N*_LN_ = 38). Correlations between protein levels of PDGF-B and urine protein (**e**; *N*_LN_ = 38). *GAPDH* glyceraldehyde-3-phosphate dehydrogenase

Glomerulonephritis of SLE patients is involved in the pathogenesis of LN. By analyzing the levels of TLR9, TGF-β1, and PDGF-B in SLE patients with LN, significant correlations were found between the mRNA expressions of TGF-β1 and PDGF-B (*p* = 0.0092, *r* = 0.61; Fig. 7a), the mRNA expressions of TLR9 and TGF-β1 (*p* = 0.0003, *r* = 0.51; Fig. 7b), and the mRNA expressions of TLR9 and PDGF-B (*p* = 0.0052, *r* = 0.44; Fig. 7c). Positive correlations were also found between the protein levels of TGF-β1 and PDGF-B (*p* < 0.0001, *r* = 0.64; Fig. 7d) in these patients. Kidney damage leads to proteinuria in SLE patients with LN. In order to further explore the possible involvement of the TLR9/TGF-β1/PDGF-B pathway in the pathogenesis of LN, the protein levels of PDGF-B homodimer were compared with the urine protein levels in SLE patients with LN. Results showed that the protein levels of PDGF-B homodimer correlated with the levels of urine protein (*p* = 0.0027, *r* = 0.49; Fig. 7e) in SLE patients with LN.

In this study, we investigated the existence of the signaling transduction pathway of TLR9/TGF-β1/PDGF-B in humans and the excessive activation of this pathway in SLE patients. Moreover, the possible involvement of the TLR9/TGF-β1/PDGF-B pathway in the pathogenesis of SLE was explored.

## Discussion

Our current study observed the presence of the TLR9/TGF-β1/PDGF-B pathway both in healthy humans and in SLE patients. Our data suggest the activation of this pathway increases in SLE patients, which may play an important role in promoting mesangial cell proliferation and contribute to glomerulonephritis of LN.

This is the first time that human samples have been used to study the presence of the TLR9/TGF-β1/PDGF-B pathway in SLE patients and healthy controls. Significant correlations between TLR9, TGF-β1, and PDGF-B were found both at the protein level and mRNA level in SLE patients and healthy controls, while no significant correlation was found between TLR9 and MCP-1, TLR9 and ISG15, or TLR9 and IFNα. Therefore, the TLR9/TGF-β1/PDGF-B pathway may be present both in SLE patients and healthy controls. Next, the possible existence of this pathway in humans observed in vivo was confirmed by cell culture experiments in vitro. CpG can significantly increase the production of TGF-β1 and PDGF-B in blood cells from both healthy controls and SLE patients. 1D11 is a neutralizing antibody of human TGF-β1. SB431542 selectively inhibits the phosphorylation of Smad3, and thereby inhibits TGF-β1-induced orientation of Smad3 in the nucleus [29]. TGF-β1 antagonists 1D11 and SB431542 significantly inhibited the production of PDGF-B, suggesting that TGF-β1 is involved in the process of TLR9-induced PDGF-B production in humans.

To further explore the activation levels of the pathway in these two groups of people, we compared the CpG effects on the production of PDGF-B in healthy controls and SLE patients; it was found that blood cells of SLE patients did produce much higher levels of TGF-β1 and PDGF-B than those of healthy controls. The TLR9/TGF-β1/PDGF-B pathway can be excessively activated in SLE patients compared to healthy controls. This could be one of the explanations for the increased levels of TLR9, TGF-β1, and PDGF-B in the peripheral blood of SLE patients compared to healthy controls. We put forward the hypothesis that higher activation levels of the TLR9/TGF-β1/PDGF-B pathway in SLE patients are associated with increased levels of TLR9, TGF-β1, and PDGF-B in the blood of SLE patients. It is worth mentioning that higher levels of protein expression of TLR9 have been reported in monocytes and different lymphocyte subsets from SLE patients compared with healthy controls by flow cytometry [30]. In addition, a significant reduction in SLEDAI after immunosuppressive treatment is associated with the decreases in TGF-β1 and PDGF-B. In agreement with these findings, increased TGF-β1 production and increased urinary levels of TGF-β1 were reported in SLE patients [31, 32]. On the other hand, increased levels of TGF-β1 found in SLE patients contradicts a study that showed lower levels of TGF-β1 in these patients [33]. Xing et al. found that TGF-β1 levels were higher in the urine of LN patients, though they were decreased in the serum of SLE patients [34]. It is not clear whether these differences in results may be due to the racial difference between the patients groups involved in the research.

Increased levels of TLR9, TGF-β1, and PDGF-B in SLE patients indicate the upregulation of this pathway. We further explored the possible involvement of this pathway in the pathogenesis of glomerulonephritis. We found that culture media of CpG-stimulated blood cells were able to stimulate mesangial cell proliferation greatly, which was an important character of glomerulonephritis. Compared to healthy controls, plasma from SLE patients can significantly increase the proliferation of mesangial cells as well. Moreover, this proliferation can be inhibited by neutralizing anti-PDGF-B antibodies. These results suggest that, at least partly because of the increased levels of PDGF-B coming from elevated TLR9/TGF-β1/PDGF-B pathway activation, plasma of SLE patients can promote mesangial cell proliferation significantly. Endogenous DNA-containing autoantibody complexes stimulate TLR9 and induce inflammation in SLE patients [35]. Therefore, in SLE patients, DNA fragments may overactivate the TLR9/TGF-β1/PDGF-B pathway to produce large amounts of TGF-β1 and PDGF-B. TGF-β1 can induce fibrosis in the kidney, while PDGF-B further stimulates the proliferation of mesangial cells. Just like in control and SLE patients, significant correlations have been observed between TLR9, TGF-β1, and PDGF-B in SLE patients with LN. Proteinuria is an important character of glomerulonephritis and LN. Furthermore, the levels of urine protein have been found to correlate with the levels of PDGF-B greatly in SLE patients with LN. Similarly, PDGF-B expression has been shown to strongly correlate with the severity of IgA glomerulonephritis, especially in terms of proliferative glomerular changes [36]. These results support the hypothesis that the TLR9/TGF-β1/PDGF-B pathway is involved in the mechanisms causing LN in SLE patients. In agreement with this, it is reported that mRNA levels of TLR9 are significantly higher in SLE patients with LN than in those without LN [37]. Our published results have proved that the TLR9 agonist induces PDGF-B production and cell proliferation through TGF-β1 signaling in mouse bone marrow macrophages [3]. It has also been reported that CpG accelerates the development of LN during the pre-active phase in NZB × NZWF1 mice [38].

TGF-β is an attractive therapeutic target, especially in chronic inflammation and tumors [39, 40]. Recombinant human anti-TGF-β1 antibody has been used in the therapy of systemic sclerosis in clinical trials [41]. Our current study demonstrates the effectiveness of neutralizing anti-PDGF-B antibodies in preventing mesangial cell proliferation in vitro. CpG-DNA/TLR9-mediated glomerulonephritis as well as the transition from inflammation to fibrosis may be inhibited by targeted inhibition of TGF-β1 and PDGF-B through antibody therapy. Cytotoxic agents and corticosteroids are standard treatments for LN with considerable morbidity and suboptimal outcomes [42]. With further clarification of the overactivated pathway of TLR9/TGF-β1/PDGF-B in LN patients, it is critical to study TLR9, TGF-β1, or PDGF-B antagonists in the prevention and treatment of LN.

There are several limitations to our study. First, we did not perform immunohistochemical staining to localize the expression of TLR9, TGF-β1, or PDGF-B in the kidneys of SLE patients. However, similar studies have been reported separately. Machida et al. found that TLR9 is not expressed in normal kidneys, but TLR9 develops in podocytes in active LN patients and disappears in remission [14]. Immunofluorescence staining of kidney biopsies showed substantial expression of TGF-β1 in LN patients [43]. The mRNA of PDGF-B and PDGF-β receptor was observed in the kidney of LN patients but not in healthy controls by in situ hybridization [44]. We are therefore confident that increased TLR9, TGF-β1, and PDGF-B can be observed in the kidney of SLE patients compared to healthy controls, and that part of this increase may be recruited from the blood. Second, the cell types that respond to CpG for the production of TGF-β1 and PDGF-B were not specified. From previous studies, TLR9 was found to be expressed mainly in plasmacytoid dendritic cells of healthy humans [45–47]. Increased levels of TLR9 have been found in different cell types (T cells, B cells, monocytes) in the blood of SLE patients compared to healthy controls [30, 48], but the exact blood cell types that express TLR9 in SLE patients have not been clearly identified. As a multiple functional cytokine, TGF-β1 can be produced by both innate and adaptive immune cells such as monocytes/macrophages, dendritic cells, and T lymphocytes. A specific cell-type culture has the significant limitation of missing the opportunity to observe the results of the cell-cell interaction under natural conditions. Therefore, whole blood assays were utilized here to yield results that may be more representative of the complex condition in vivo. On the other hand, similar results have been observed in the culture experiments utilizing monocytes isolated from the blood of SLE patients compared to that utilizing whole blood cells (see Additional file 2: Figure S1). Our studies focus on proving that the TLR9/TGF-β1/PDGF-B pathway found in mice also exists in humans; we provide new evidence that TLR9 signals to induce TGF-β1 and PDGF-B, which are critical mediators of glomerulonephritis. Finally, this study supports the presence and overactivation of the TLR9/TGF-β1/PDGF-B pathway in SLE patients, but its link to the pathogenesis of LN requires further supporting evidence. We cannot make the conclusion that TLR9, TGF-β1, or PDGF-B antagonists can be used as treatments for LN, though we hypothesize that TLR9, TGF-β1, and PDGF-B may serve as new therapeutic targets for SLE. In agreement with this hypothesis, Fukasawa et al. have successfully treated chronic progressive nephritis of rats with anti-TGF-β antibody by inhibiting Smad/TGF-β signaling [49]. Moreover, the tyrosine kinase inhibitor of the PDGF receptor, imatinib, has been reported to ameliorate LN in mouse models including NZB/W lupus mice and MRL/lpr mice [50, 51].

## Conclusions

Our results show the TLR9/TGF-β1/PDGF-B pathway exists in humans and that it is overactivated in the peripheral blood of SLE patients. Further analysis on the involvement of this pathway in the pathogenesis of glomerulonephritis may provide important additional information to assist the physician in understanding the underlying cause of LN, which may lead to development of TLR9, TGF-β1, or PDGF-B antagonists to prevent the development of renal fibrosis and renal failure in LN.

## Additional files

Additional file 1: Table S1. Clinical data of the study subjects. (XLS 23 kb)

Additional file 2: Figure S1. CpG induces upregulation of TGF-β1 and PDGF-B in monocytes in vitro. Isolated monocytes from healthy controls (A and B; *N*_Control_ = 8) and SLE patients (C and D; *N*_SLE_ = 7) were stimulated with or without 500 nM CpG for 24 h, and then mRNA expression of TGF-β1 and PDGF-B were detected by qPCR. Multiple rates of mRNA expression of TGF-β1 (E) and PDGF-B (F) in healthy controls (*N*_Control_ = 8) and SLE patients (*N*_SLE_ = 7) were calculated as CpG/Media. The results are presented as mean and SEM. (PPTX 510 kb)

## Abbreviations

ACR: American College of Rheumatology
CpG: Hypomethylated CpG oligonucleotide-motif DNA
DMSO: Dimethylsulfoxide
ECM: Extracellular matrix
ELISA: Enzyme-linked immunosorbent assay
FBS: Fetal bovine serum
GAPDH: Glyceraldehyde-3-phosphate dehydrogenase
HCQ: Hydroxychloroquine
IFN: Interferon
ISG: Interferon-stimulated gene
LN: Lupus nephritis
MCP: Monocyte chemoattractant protein
MMF: Mycophenolate mofelil
MTT: 3-(4,5-dimethylthiazol-2-yl)-2,5-diphenyl-2H-tetrazolium bromide
PBMC: Peripheral blood mononuclear cell
PDGF-B: Platelet-derived growth factor B
qPCR: Real-time quantitative polymerase chain reaction
SLE: Systemic lupus erythematosus
SLEDAI: SLE Disease Activity Index
TGF: Transforming growth factor
TLR: Toll-like receptor
