# Supplementary material for: Excessive activation of the TLR9/TGF-β1/PDGF-B pathway in the peripheral blood of patients with systemic lupus erythematosus
Source: Arthritis Res Ther. 2017 Mar 29;19:70. doi: 10.1186/s13075-017-1238-8 (PMC5372299; doi:10.1186/s13075-017-1238-8)
Supplement: Supplementary file 2 — CpG induces upregulation of TGF-β1 and PDGF-B in monocytes in vitro. Isolated monocytes from healthy controls (A and B; N Control = 8) and SLE patients (C and D; N SLE = 7) were stimulated with or without 500 nM CpG for 24 h, and then mRNA expression of TGF-β1 and PDGF-B were detected by qPCR. Multiple rates of mRNA expression of TGF-β1 (E) and PDGF-B (F) in healthy controls (N Control = 8) and SLE patients (N SLE = 7) were calculated as CpG/Media. The results are presented as mean and SEM. (PPTX 510 kb) [file 13075_2017_1238_MOESM2_ESM.pptx]

## Slide 1
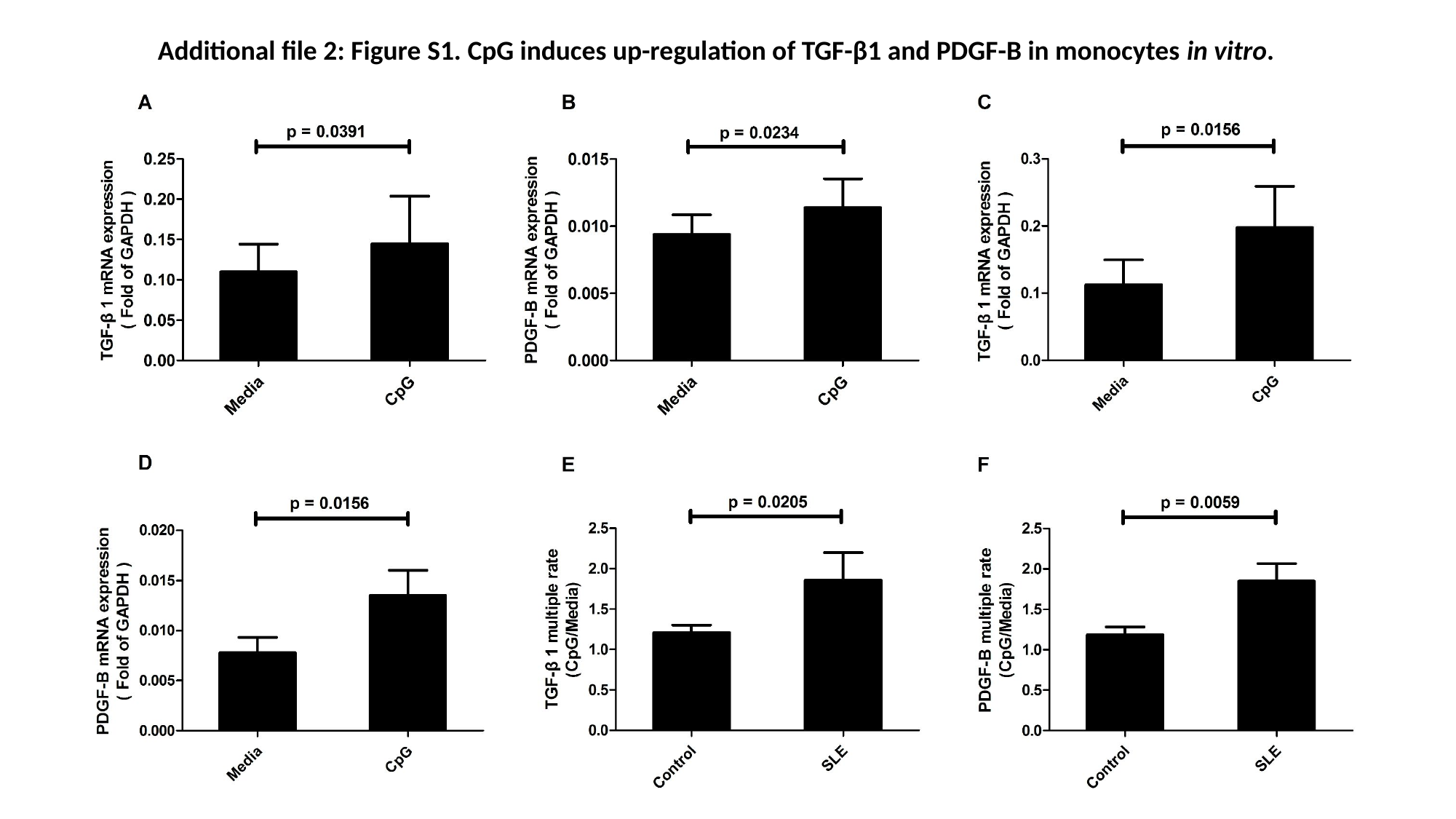

Additional file 2: Figure S1. CpG induces up-regulation of TGF-β1 and PDGF-B in monocytes in vitro.
